# American Academy of Dental Science—Resolutions of Respect

**Published:** 1891-08

**Authors:** 


*Secretary.*

---

### AMERICAN ACADEMY OF DENTAL SCIENCE—RESOLUTIONS OF RESPECT.

At the monthly meeting of the American Academy of Dental Science, held in Boston, June 3, 1891, the Corresponding Secretary announced the death of Edward Maynard, A.M., M.D., D.D.S., of Washington, D. C.; James W. White, A.M., M.D., D.D.S., of Philadelphia, Pa.; and William H. Atkinson, A.M., M.D., D.D.S., of New York.

Resolutions of respect to their memory were unanimously adopted, deploring their departure, and expressive of their high character and great professional and private worth, and of their long and earnest efforts and labors in behalf of dentistry and dental education as writers, teachers, and practitioners, who have contributed so largely to make the profession worthy of its present advanced position, and tendering the sympathy of the Academy to their widows and families in their bereavement and sorrow.

These resolutions were ordered to be placed *in memoriam* upon the records of the Academy, and copies sent to the families of the deceased.

